# Morphological and Molecular Characterization of Tick Species Infesting Cattle in South Africa

**DOI:** 10.3390/vetsci11120638

**Published:** 2024-12-10

**Authors:** Tsireledzo Goodwill Makwarela, Nkululeko Nyangiwe, Tracy Madimabi Masebe, Appolinaire Djikeng, Lucky Tendani Nesengani, Rae Marvin Smith, Ntanganedzeni Olivia Mapholi

**Affiliations:** 1 College of Agriculture & Environmental Sciences, University of South Africa, Private Bag X6, Roodepoort 1710, South Africa; nyangn@unisa.ac.za (N.N.); masebtm@unisa.ac.za (T.M.M.); a.djikeng@cgiar.org (A.D.); smithrm@unisa.ac.za (R.M.S.); maphon@unisa.ac.za (N.O.M.); 2 Department of Nature Conservation, Tshwane University of Technology, Staatsartillerie Rd, Pretoria West, Pretoria 0183, South Africa; 3 International Livestock Research Institute (ILRI), P.O. Box 30709, Nairobi 00100, Kenya

**Keywords:** 16S rRNA sequencing, cattle ticks, phylogenetic analysis, tick identification, tick-borne diseases, South Africa

## Abstract

Ticks are a significant threat to livestock globally, with certain species displaying distinct host preferences at various developmental stages. Accurate species-level identification is essential for studying tick populations, implementing control strategies, and understanding disease dynamics. This study evaluated ticks infesting cattle across six provinces in South Africa using morphological and molecular methods. Ticks were preserved, examined morphologically using an Olympus Digital Camera Microscope, and identified using the 16S rRNA gene. The study identified four genera, namely *Amblyomma*, *Hyalomma*, *Ixodes*, and *Rhipicephalus*, comprising 15 ixodid species. *Amblyomma hebraeum* was the most prevalent species, with an infestation rate of 54.4%. Molecular analysis revealed genetic relationships among tick species, with genetic distances ranging from 0.00 to 0.13, and phylogenetic analysis clustered species into distinct genera with high bootstrap support. Principal Component Analysis highlighted clear genetic relatedness among species. These findings enhance our understanding of tick diversity, morphology, and distribution in South Africa’s cattle populations, emphasizing their economic significance. The study provides critical baseline data for monitoring and developing effective strategies to manage tick-borne diseases, ensuring improved livestock health and productivity.

## Simple Summary

Ticks significantly impact livestock by causing diseases that affect animal health and reduce productivity. This study examined ticks infesting cattle across six provinces in South Africa, aiming to identify and understand the diversity of tick species and their potential to transmit diseases. Over 3500 ticks were collected, preserved, and analysed using a microscope and identification DNA sequencing. The findings revealed 15 species of ticks, with the most common being *Amblyomma hebraeum*, which accounted for more than half of all ticks found. The genetic analysis confirmed their identification and highlighted their diversity. This research is vital for understanding the tick species present, and their distribution, in South Africa. Regular monitoring of tick populations and using targeted control measures can help protect livestock from the harmful effects of ticks and improve cattle health and productivity. These findings are valuable to farmers, veterinarians, and researchers, offering practical knowledge to mitigate economic losses caused by tick infestations. Effective management of ticks will also enhance food security and the sustainability of livestock farming in South Africa.

## 1. Introduction

Ticks are invertebrate ectoparasites classified within the order *Ixodida*, part of the subclass *Acari* in *Arachnida* [1]. The *Acari* subclass contains multiple orders, with *Ixodida* being the one specifically comprising ticks. Approximately 900 tick species have been reported worldwide [2], and *Ixodida* are divided into three families: *Argasidae* (soft ticks), *Ixodidae* (hard ticks), and *Nuttalliellidae* [3,4]. The *Argasidae* family has two subfamilies, *Argasinae* (n = 56 species) and *Ornithodorinae* (n = 114 species) (n = 114 species) [5], while *Nuttalliellidae* contains a single species, *Nuttalliella namaqua*, which exhibits characteristics intermediate between the *Argasidae* and *Ixodidae* families [6]. The *Ixodidae* family, commonly referred to as hard ticks, is the largest family within *Ixodida*. It is further divided into two major groups: *Prostriata*, which includes the single genus *Ixodes* (about 243 species), and *Metastriata*, which encompasses four subfamilies: *Amblyomminae* (n = 125 species), *Haemaphysalinae* (n = 147 species), *Hyalomminae* (n = 22 species), and *Rhipicephalinae* (n = 119 species) [7,8].

Hard ticks (*Ixodidae*) feed on the blood of terrestrial vertebrates, including mammals, reptiles, and birds [9,10,11]. They are significant vectors of a wide range of pathogens, including bacteria, viruses, protozoans, and helminths, which cause diseases in both animals and humans, particularly in tropical and subtropical regions [12,13,14]. In livestock, tick infestations can lead to severe economic losses by causing paralysis, blood loss, reduced body weight, decreased milk production, and, in some cases, death if untreated [15,16]. During feeding, they inject saliva containing toxins that can trigger allergic reactions, anemia, and skin irritation in the host [12,17]. Furthermore, many pathogens transmitted by ticks target the host’s blood cells, compounding the severity of infections [16]. Ticks also produce antioxidant enzymes in their gut to counter oxidative stress, but these enzymes can induce cellular damage and inflammation in their hosts, complicating immune responses and exacerbating the impact of tick infestations on cattle [18].

Farmers primarily use acaricides for tick control [17]. However, ticks can develop resistance, environmental degradation, tainted meat and milk, and high costs [19]. Therefore, regular monitoring of ticks for acaricide resistance is crucial [20]. Traditional tick identification relies on morphological characteristics viewed under light microscopy [21]. However, this method makes it difficult to identify immature ticks and damaged specimens [22]. Modern techniques use molecular markers targeting nuclear and mitochondrial DNA, allowing for more accurate taxonomy and phylogenetic analysis [23]. Markers such as Cytochrome Oxidase subunit I (COI), Cytochrome Oxidase subunit III (COIII), 12S, and 16S rDNA genes are used for genetic diversity analysis [24]. The 16S rDNA gene is particularly useful for identifying and analysing tick phylogeny [25,26]. Due to its conserved nature, COI provides additional phylogenetic resolution and is effective for examining deeper evolutionary relationships [27,28]. This combination has enhanced the understanding of genetic differentiation among populations of various marine and terrestrial organisms [27,29].

The 12S rRNA gene, though less commonly used than *COI*, is valuable for studying population structure and genetic variability. It complements *COI* analyses and helps reveal genetic differentiation crucial for conservation efforts [30,31]. The intergenic spacer (ITS) regions are less conserved than coding regions and highly variable. They are increasingly used for genetic diversity studies, especially in populations with significant evolutionary divergence. These regions are also useful for distinguishing closely related populations [32,33].

Based on the literature, tick species such as *Amblyomma hebraeum* Koch, 1844; *Amblyomma variegatum* Fabricius, 1794; *Hyalomma rufipes* Koch, 1844; *Hyalomma truncatum* Koch, 1844; *Ixodes rubicundus* Neumann, 1904; *Rhipicephalus decoloratus* Koch, 1844; *Rhipicephalus microplus* Canestrini, 1888; *Rhipicephalus appendiculatus* Neumann, 1901; and *Rhipicephalus evertsi evertsi* Neumann, 1897 infest cattle in South Africa [34,35,36]. Identifying these ticks is crucial for developing effective control measures and reducing economic losses in the livestock industry. This study aimed to determine the morphological and genetic characteristics of economically significant ticks infesting cattle, providing insights into their diversity, distribution, and potential implications for tick-borne diseases.

## 2. Materials and Methods

### 2.1. Ethical Approval

Ticks were collected from natural grazing communal cattle during their dipping time. Staff members from the Department of Agriculture at the targeted study sites assisted with removing the ticks while ensuring that the cattle concerned remained unharmed. Ethical approval was obtained from the UNISA-CAES Animal Research Ethics Committee (Ref #: 2022/CAES-AREC/036) and DAFF (Ref: 12/11/1/1/23 (1466AC)).

### 2.2. Study Area and Sample Collection

**Figure 1:**
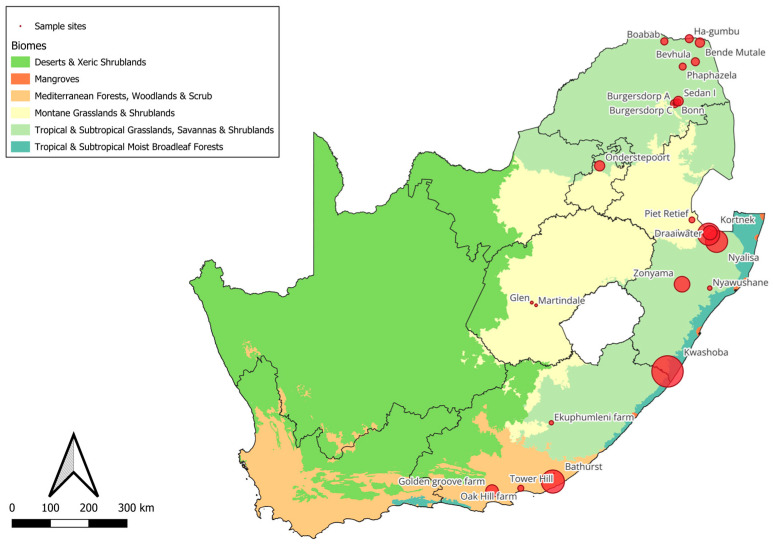
A map of South Africa, highlighting the study sites from which hard ticks were collected. The green letters denote the specific provinces concerned, whereas the red dots signify the locations of the 21 dip tanks from which the collection of ticks occurred: n = 9 Limpopo (LP), n = 1 Gauteng (GP), n = 1 Mpumalanga (MP), n = 4 KwaZulu-Natal (KZN), n = 2 Free State (FS), and n = 4 Eastern Cape (EC), where “n” represents the number of localities per province.

A total of 3514 tick specimens were collected during the summer seasons from September to December in 2021 and 2022. The collection sites included twenty-one dip tanks across six South African provinces as shown in Figure 1, encompassing diverse climatic zones. Specifically, ticks were collected in semi-arid regions: Limpopo (LP, n = 909 ticks), Mpumalanga (MP, n = 137 ticks), and Eastern Cape (EC, n = 643 ticks); in tropical wet regions: KwaZulu-Natal (KZN, n = 1562 ticks); and in dry regions: Gauteng (GP, n = 167 ticks) and Free State (FS, n = 96 ticks). Cattle were randomly selected, ensuring to collect from a minimum of two and a maximum of ten cattle per farmer or per household. This approach aimed to capture representative tick populations from diverse environmental conditions. Ticks were collected using the Patch sampling method, which involves sampling specific predilection sites by hand-picking, as described by Mooring and McKenzie [37]. The presumption was that the number of ticks collected from these sites would indicate the relative degree of infestation [37]. Various predilection sites, such as ears, neck, withers, dewlap, perineal region (udder in females and testes in males along with the perineum region), and tail, were inspected for the presence of ticks. Ticks were sampled during the collection period, with an average of approximately 167 ticks per dip tank. Upon collection, ticks were preserved in 70% ethanol to maintain specimen integrity [38]. The preserved ticks were then washed in distilled water and air-dried on filter paper. Morphological examination was conducted using an Olympus Digital camera Microscope (model DP74) to capture high-resolution images of each specimen. Identification was based on morphological characteristics using established tick identification keys specific to the region or species, as referenced in [34,39].

### 2.3. Morphological Examination

Ticks were transported to the Eureka Building at the Unisa Science Campus in Florida, Gauteng, South Africa, for morphological identification. They were identified at the species level under a stereomicroscope (Carl Zeiss Microscopy GmbH, Stemi 508), analysing morphological characteristics using the taxonomic keys of [34]. Ticks were categorized into the genera *Hyalomma*, *Rhipicephalus*, *Haemaphysalis*, *Amblyomma*, and *Ixodes* based on morphology.

Additionally, An Olympus microscope at x400 magnification was used to identify *Rhipicephalus microplus* and *Rhipicephalus decoloratus* ticks at the species level, as hypostome dentition is a key morphological feature for distinguishing species within this genus.

### 2.4. DNA Extraction and PCR Amplification

For molecular identification, DNA was extracted from 255 individual ticks using the E.Z.N.A Tissue DNA Kit (Omega Bio-Tek, Norcross, GA, USA), following the manufacturer’s protocol [40]. The 16S rRNA gene was amplified using PCR with the universal primers 16S + 1 (5′-CTGCTCAATGATTTTTTAAATTGCTGTGG-3′) and 16S − 1 (5′-CCGGTCTGAACTCAGATCAAGT-3′) [41]. The reaction mixture for each amplification had a total volume of 25 μL and included the following components: 12.5 μL of 1x Premix Ex Taq (Takara Bio Europe SAS, Saint-Germain-en-Laye, France), 2.5 μL of both forward and reverse primers (0.5 µM), 2 μL of the DNA template, and 8 μL of distilled water (dH_2_O). The thermal cycler was programmed for the amplification process as follows: initial denaturation at 95 °C for 5 min, followed by 40 cycles consisting of denaturation at 95 °C for 45 s, annealing at 55 °C for 60 s, extension at 72 °C for 90 s, and a final extension step at 72 °C for 5 min. A negative control containing water instead of DNA was included in the amplification, while a DNA ladder was used to estimate the size of the amplified products. To confirm successful amplification, the resulting amplicons of approximately 410 bp were visualized using a 2% agarose gel using ethidium bromide dye and the Bio-Rad Gel Doc XR+ UV Gel System. Subsequently, the amplified products were sent for sequencing using the Sanger sequencing method at the Central Analytical Facilities of Stellenbosch University in South Africa.

### 2.5. Sequencing and Phylogenetic Analysis

PCR products were purified using the QIAquickÔ PCR Purification Kit (Qiagen, Hilden, Germany) and sequenced using an ABI PRISM 3730xL Genetic Analyzer (Thermo Fisher Scientific Company, Waltham, MA, USA). Sequence alignment and editing were performed using MEGA11 software [42]. Phylogenetic trees were constructed using the Maximum Likelihood method based on the Tamura-Nei model [43]. Bootstrap analysis with 1000 replicates was used to assess the robustness of the phylogenetic trees [44]. Sequences were deposited in NCBI and GenBank; the accession number(s) are PP789312–PP789494.

### 2.6. Genetic Distance and Principal Component Analysis

Intraspecific genetic distances were calculated using Kimura 3-parameter (K3P) model [45]. Principal Component Analysis (PCA) was employed to demonstrate the clustering of different tick genera. PCA was performed using the “prcomp” function in R ver. 4.1.

### 2.7. Data Analysis and Interpretation

The sequence data were analysed to confirm the results of the morphological identification and the distribution of different tick species. The results were correlated with climatic and geographic data to identify patterns in tick distribution. Moreover, statistical analyses were performed using Statistical Package for the Social Sciences (SPSS) Statistics 29.0 (IBM), with significance set at *p* < 0.05.

## 3. Results

The tick species distribution across South African provinces is summarized in Table 1. The representation highlights distinct patterns of species prevalence and regional diversity, with *A. hebraeum* and *R. microplus* being dominant in tropical regions like KwaZulu-Natal and Limpopo, while species such as *H. silacea* and *R. gertrudae* show restricted distributions. The results indicate KwaZulu-Natal as the province with the highest tick diversity and abundance, followed by Limpopo and the Eastern Cape.

**Figure 2:**
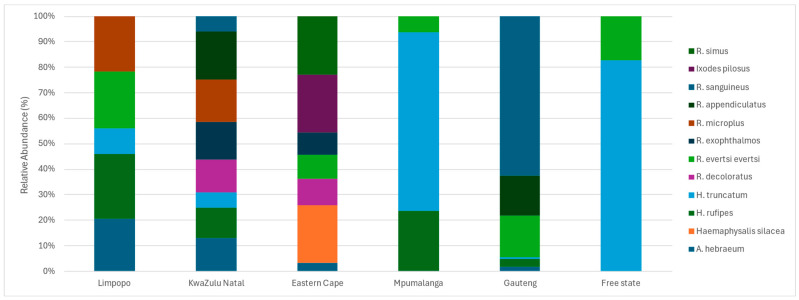
Relative abundance of the five most common tick species across the surveyed locations. Each bar represents a location, with segments illustrating the proportional representation of each species.

In Figure 2, the bar graph illustrates the relative abundance and diversity of tick species across six locations, normalized to 100%. The data reveal clear geographical variation in species composition. Limpopo is dominated by *Rhipicephalus appendiculatus* and *Rhipicephalus microplus*, which together constitute a significant proportion of the tick population. In KwaZulu-Natal, the tick community is more diverse, with contributions from *Ixodes pilosus* and *Rhipicephalus sanguineus*, among others. The Eastern Cape features a balanced distribution of species, including *Amblyomma hebraeum* and *Haemaphysalis silacea*. In Mpumalanga, the tick population is largely composed of *Rhipicephalus microplus*, showing limited diversity. Gauteng displays a relatively balanced tick community, including *Rhipicephalus evertsi evertsi* and *Rhipicephalus exophthalmos*. Lastly, Free State exhibits low diversity, with *Rhipicephalus sanguineus* dominating.

**Figure 3:**
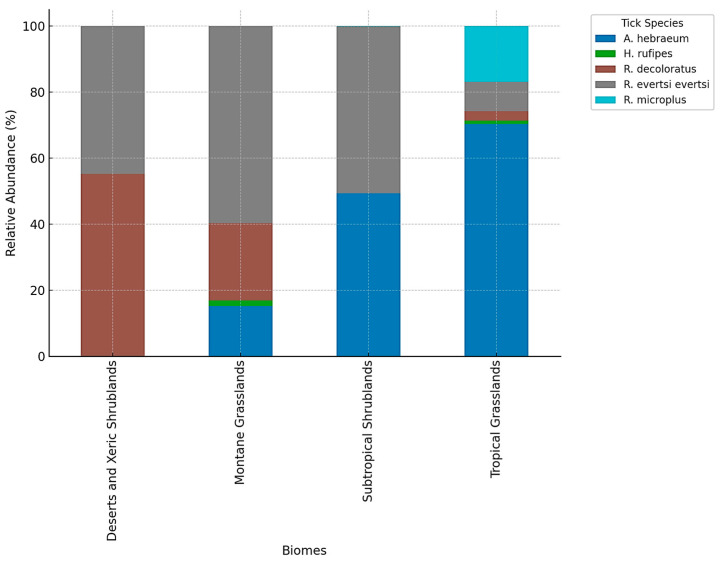
Relative abundance of the most common tick species (*A. hebraeum*, *H. rufipes*, *R. decoloratus*, *R. evertsi evertsi*, and *R. microplus*) across four biomes in South Africa. The chart highlights the ecological adaptations of tick species to distinct biomes, with *A. hebraeum* and *R. microplus* prevalent in Tropical Grasslands, *R. decoloratus* dominating Deserts and Xeric Shrublands, and a more balanced distribution in Montane Grasslands and Subtropical Shrublands.

The bar chart in Figure 3 illustrates the relative abundance of the five most common tick species (*A. hebraeum*, *H. rufipes*, *R. decoloratus*, *R. evertsi evertsi*, and *R. microplus*) across four distinct biomes in South Africa: Deserts and Xeric Shrublands, Montane Grasslands, Subtropical Shrublands, and Tropical Grasslands. The distribution patterns reveal significant ecological adaptations of tick species to specific environmental conditions. In Tropical Grasslands, *A. hebraeum* and *R. microplus* dominate, reflecting their preference for warm, humid environments. Subtropical Shrublands are characterized by the prevalence of *R. decoloratus* and *R. evertsi evertsi*, which thrive in moderately warm and less humid conditions. Montane Grasslands, with their cooler and semi-arid climate, exhibit a more balanced distribution of *R. decoloratus*, *H. rufipes*, and *R. evertsi evertsi*, showcasing their adaptability to a wider range of environmental conditions. In contrast, Deserts and Xeric Shrublands are primarily dominated by *R. decoloratus*, a species well-suited to arid conditions.

### 3.1. Morphological Characterization

**Figure 4:**
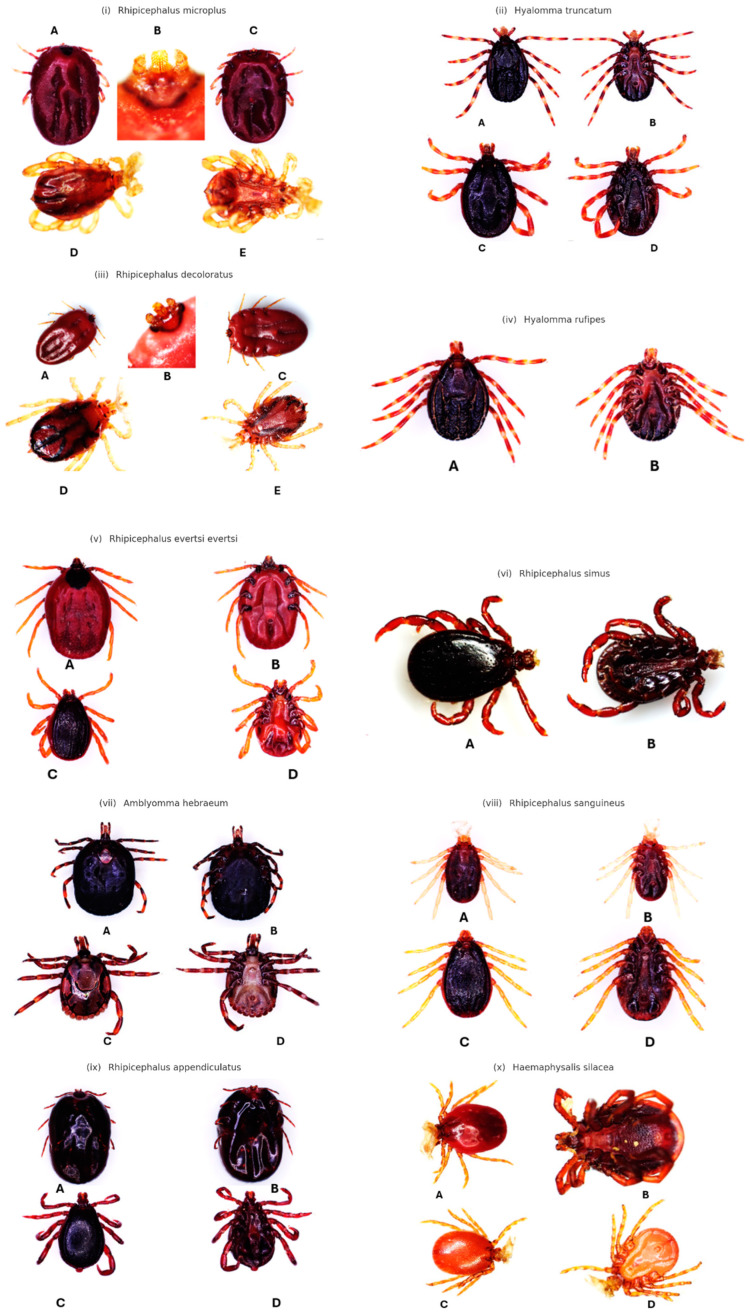
Morphological characteristics of tick species infesting cattle in South Africa. This figure illustrates the morphological characteristics of various tick species infesting cattle in South Africa: (**i**) *R. microplus*, (**ii**) *H. truncatu*m, (**iii**) *R. decoloratu*s, (**iv**) *H. rufipes*, (**v**) *R. evertsi evertsi*, (**vi**) *Rhipicephalus simus*, (**vii**) *A. hebraeum*, (**viii**) *Rhipicephalus sanguineus*, (**ix**) *R. appendiculatus*, and (**x**) *Hae. silacea*.

The morphological features in Figure 4 of the tick species identified in this study were distinct and crucial for accurate identification. *A. hebraeum* was characterized by long mouthparts, prominent eyes, and coloured rings on reddish legs. Females exhibited a wider scutum posterior angle, convex sides, and pale leg rings, while males displayed variations in conscutum coloration. *R. evertsi evertsi* had medium-length hypostomes and palps, a dark conscutum in males with faint, curved adanal plates, and convex, beady eyes. Females of this species had unevenly arranged orange legs. *R. decoloratus* featured visible dark caeca through its pale yellow and translucent scutum, short mouthparts, and hypostome teeth arranged in 3 + 3 rows. In *R. microplus*, the reddish-brown scutum, short palps, and hypostome teeth arranged in 4 + 4 rows were notable characteristics. *R. appendiculatus* was identified by its short hexagonal shape in females and a visible anterior process of coxa I in males. Females had slightly convex eyes and a shallow V-shaped vaginal pore, whereas males had broader cervical fields with sharply elevated edges. *Rhipicephalus sanguineus* females possessed a wide U-shaped genital aperture with spiral plates, while males exhibited deeply wrinkled back grooves and thin trapezoid adanal plates. *Rhipicephalus simus* females had a dark, smooth, and glossy scutum with punctations delineating the cervical groove. *H. rufipes* was identified by a dark scutum with whitish rings on its legs, scapular grooves, and a broad V-shaped posterior lip of the vaginal opening. *H. truncatum* featured a dark brown, narrow, and smooth conscutum with few punctuations, pale ring coloration on legs, and a wide U-shaped genital aperture in females. Lastly, *Hae. silacea* displayed a broadly ovate body in males, triangular scapulae with rounded apices, a U-shaped genital aperture in females, and densely distributed small punctations on the conscutum. These detailed morphological characteristics were essential for the identification and understanding of the tick species infesting cattle in South Africa, aiding in the development of effective control measures against tick-borne diseases.

### 3.2. Maximum Likelihood Estimate of Substitution Matrix

Table 2 presents the frequency of base substitutions per site among sequences. Substitution patterns and rates were estimated under the Tamura [46] model. The substitution rates between different nucleotides indicate that transitions (substitutions between purines or pyrimidines) generally occurred at higher rates than transversions (substitutions between a purine and a pyrimidine). For instance, the transition rates from adenine (A) to guanine (G) and from cytosine (C) to thymine (T) were particularly frequent. The dataset, comprising 184 nucleotide sequences and 508 positions, revealed significant mutational bias or selective pressure favouring these substitutions. The computed tree topology and Maximum Likelihood value (−2708.700) reflected the sequences’ evolutionary relationships, with the higher transition rates, particularly C to T, suggesting a mutational bias or selective pressure favouring these substitutions, underscoring significant patterns in the evolutionary dynamics of the sequences analysed.

### 3.3. Molecular Classification of Tick Taxa

**Figure 5:**
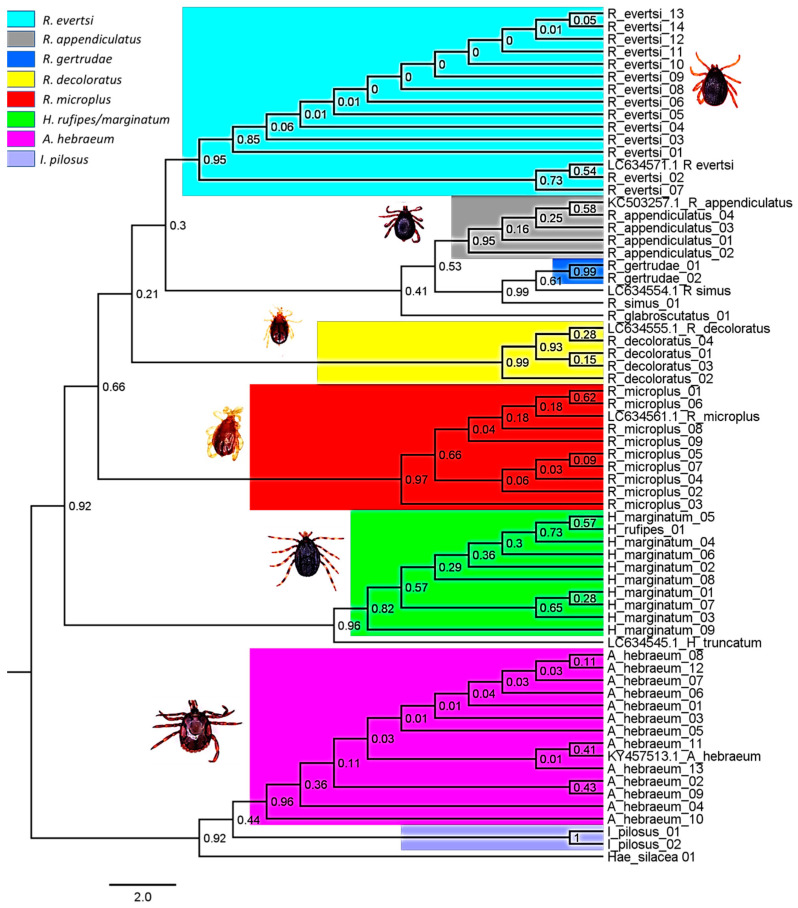
A phylogenetic tree illustrating relationships among cattle tick species from various genera. The analysis includes reference sequences from GenBank, totalling sixty-eight nucleotide sequences representing all species across study sites. Using MEGA11 [47] with the Tamura 3-parameter model [46], the evolutionary history was inferred, and the resulting tree was adjusted and visualized using Figtree v1.4.4 and Gimp 2.0.

A Maximum Likelihood phylogenetic tree (Figure 5) was constructed using MEGA11 software, incorporating 60 representative nucleotide sequences from various tick species, including *R. appendiculatus* (n = 4), *R. microplus* (n = 9), *R. decoloratus* (n = 4), *I. pilosus* (n = 2), *Hae. silacea* (n = 1), *A. hebraeum* (n = 12), *Hyalomma marginatum/rufipes* (n = 9), *Rhipicephalus simus* (n = 1), *R. glabroscutatus* (n = 1), *H. rufipes* (n = 1), and *R. evertsi evertsi* (n = 14). Additionally, seven reference sequences in Table 3 were obtained from GenBank, identified by their accession numbers: KC503257.1, KY457513.1, LC634555.1, LC634561.1, LC634554.1, LC634545.1, and LC634571.1. For certain species, such as *Hae. silacea*, *R. exophthalmos*, *R. glabroscutatus*, *R. sanguineus*, and *I. pilosus*, there were no reference nucleotide sequences available in the GenBank database at the time of the study. The phylogenetic tree and BLAST analysis results (Table 3) revealed genetic relationships among tick species, with bootstrap values supporting the robustness of inferred relationships. The Maximum Likelihood phylogenetic tree showed that species within the same genus tended to cluster together with high bootstrap values of approximately 90%, indicating strong phylogenetic associations.

### 3.4. Estimates of Evolutionary Divergence Between Sequences

The genetic distance matrix in Table 4 indicates significant genetic diversity among the sequences. The number of base substitutions per site from between sequences, with standard error estimates shown above the diagonal, was obtained by a bootstrap procedure (1000 replicates). Analyses were conducted using the Maximum Composite Likelihood model. This analysis involved 48 nucleotide sequences with all ambiguous positions removed for each sequence pair (pairwise deletion option), resulting in a total of 508 positions in the final dataset. The genetic distances, based on 16S rRNA gene sequences, ranged from 0.00 to 0.13, reflecting the genetic variability among tick species. Lower values indicated high genetic similarity, while higher values suggested greater divergence. The highest genetic distance observed was 0.13, reflecting the most significant divergence within the dataset. Overall, most genetic distances fell between 0.00 and 0.03, suggesting that the tick specimens were generally closely related.

### 3.5. Phylogenetic Trees

Figure 5 shows phylogenetic trees with distinct clusters corresponding to different tick genera. The Amblyomma clade included species such as *A. hebraeum*, indicating close evolutionary relationships. The *Rhipicephalus* clade featured multiple species, including *Rhipicephalus microplus*, *R. appendiculatus*, and *R. evertsi evertsi*, which clustered together, demonstrating strong phylogenetic associations. The *Ixodes* clade included *Ixodes ricinus*, forming another distinct cluster. These clusters were well-supported by high bootstrap values, indicating robust phylogenetic relationships. The clustering pattern reflected the taxonomic classifications and evolutionary relationships among the tick species, highlighting the genetic distinctiveness of each genus.

### 3.6. Population Structures Using PCA

**Figure 6:**
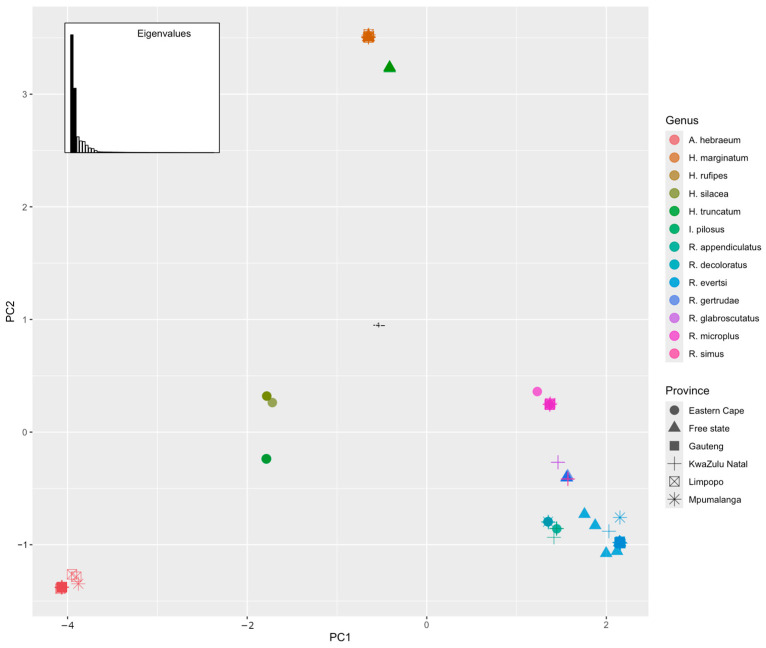
Principal Component Analysis (PCA) of various hard tick species from different provinces in South Africa. Each dot represents a population of a specific tick species, with colours distinguishing the species: *R. appendiculatus* (dark green), *R. microplus* (brown), *R. decoloratus* (light green), *R. pilosus* (maroon), *H. silacea* (green), *A. hebraeum* (yellow), *H. marginatum* (dark blue), *R. simus* (pink), and *R. evertsi* (light blue). Symbols represent provinces: Eastern Cape, Free State, Gauteng, Kwazulu Natal, Limpopo, and Mpumalanga. Closer dots indicate greater genetic similarity, while those farther apart denote genetic distinctiveness. The PCA reveals distinct genetic clusters influenced by species and geographical locations.

The Principal Component Analysis (PCA) biplot in Figure 6, executed using the adegenet package in R version 4.4.4, utilizes genetic distance data extracted from sequence alignments derived from single nucleotide polymorphisms (SNPs) to reveal distinct genetic clusters among various tick species populations. The *x*-axis (PC1) and *y*-axis (PC2) explain the majority of the genetic variance, as confirmed by the eigenvalues plot, which highlights the significant contribution of the first two principal components.

Distinct clustering patterns are evident among the species populations. *A. hebraeum*, located in the bottom-left quadrant, exhibits unique genetic traits distinct from other species, indicated by its negative correlation with both PC1 and PC2. Conversely, *I. pilosus*, positioned near the centre-left, shows moderate negative correlations with PC1 and PC2, indicating a mix of shared and unique genetic traits. *Hae. silacea*, found near the centre-right, has positive correlations with both PC1 and PC2, suggesting it possesses a balanced combination of common and unique genetic characteristics. The clusters of *H. marginatum* and *H. truncatum* in the top-right quadrant highlight shared but distinct genetic traits, while *R. microplus*, also in the top-right, shows strong positive correlations with both PC1 and PC2, indicating significant unique genetic features. The clusters of *R. appendiculatus* and *R. evertsi* in the right-middle suggest shared genetic traits with slight differences, and *R. gertrudae*, *R. simus*, and *R. decoloratus* overlap in the lower right quadrant, indicating high genetic similarity with minor variations.

## 4. Discussion

### 4.1. Distribution and Ecological Roles of Cattle Tick Species in South Africa

The distribution of various tick species in South Africa provides valuable insights into their ecological roles, host preferences, and potential risks to livestock and wildlife health. Comparing our findings with previous research highlights both consistent patterns and novel discoveries, emphasizing the importance of localized studies in understanding tick ecology and epidemiology.

The study confirmed *Amblyomma hebraeum* as the most prevalent tick species, particularly in KwaZulu-Natal and Limpopo, with smaller populations in the Eastern Cape. This aligns with findings from Mapholi, Banga [48] and Horak, Boshoff [49], which identified *A. hebraeum* as dominant in savanna and grassland habitats in the eastern regions of South Africa. However, our research also noted its adaptability to semi-arid regions, suggesting greater ecological flexibility than previously documented. Its strong association with cattle as a host and its role as a vector for *Theileria parva* underline its critical importance in livestock health and disease management. *Haemaphysalis silacea* was found exclusively in the Eastern Cape, consistent with its highly localized distribution documented in earlier studies [49]. Its preference for dense vegetation and reliance on small mammals as hosts are well-established. The study, however, contributes new observations, such as unique setae patterns that aid in its identification. These findings emphasize the importance of targeted research on less-studied species to better understand their ecological roles and potential contributions to disease transmission. The distribution of *Hyalomma truncatum* and *Hyalomma rufipes* in the study closely mirrors previous research, with both species demonstrating broad distributions across South Africa. *Hyalomma truncatum* was most common in semi-arid regions, while *Hyalomma rufipes* showed a preference for more humid environments. These results corroborate studies by Fournier, Gouriet [50] and Esmaeel, Hussain [51]. However, our research highlights the overlapping distributions of these species, raising questions about interspecies competition and shared ecological niches. Additionally, the adaptability of *H. rufipes* to wetter environments extends our understanding of its ecological versatility.

The detection of *Ixodes pilosus* primarily in the Eastern Cape and Mpumalanga extends its known host range beyond small mammals and birds, as reported by Horak, Boshoff [49]. Our findings suggest that this species demonstrates a degree of host flexibility, having been observed in cattle. This broader host association raises new questions about its role in pathogen transmission and ecological dynamics, warranting further investigation. The *Rhipicephalus* genus exhibited significant diversity and adaptability, with several species showing distinct ecological preferences. *Rhipicephalus decoloratus* and *Rhipicephalus microplus* were widely distributed, with the former being more common in drier regions and the latter in humid areas, consistent with findings by Mapholi, Banga [48]. The study highlights the high prevalence of *R. microplus* in KwaZulu-Natal and Limpopo, reinforcing its economic impact on livestock due to its role in transmitting *Babesia bovis* and *Anaplasma marginale*. Additionally, *Rhipicephalus appendiculatus* was primarily found in KwaZulu-Natal, confirming its established role in the transmission of *Theileria parva*. Less common species like *Rhipicephalus gertrudae* and *Rhipicephalus glabroscutatus* were observed in localized regions, with the former restricted to the Free State and the latter to KwaZulu-Natal. These findings align with the specific ecological niches described by Horak, Boshoff [49]. Meanwhile, *Rhipicephalus sanguineus*, often associated with domestic dogs in urban areas, was found in peri-urban regions, suggesting its potential role in zoonotic disease transmission where human–animal interactions are frequent.

Our findings provide several new insights into tick distribution in South Africa. The broader host range of *Ixodes pilosus* highlights its ecological flexibility, potentially influencing its role as a vector. The adaptability of *Amblyomma hebraeum* and *Hyalomma rufipes* to diverse environmental conditions suggests these species may pose increasing challenges under changing climatic conditions. Additionally, the restricted distributions of less-studied species like *Haemaphysalis silacea* and *Rhipicephalus gertrudae* underscore the importance of localized research in capturing regional biodiversity.

### 4.2. Morphological Characterization of Tick Species Infesting Cattle in South Africa

This study presents a detailed morphological and molecular analysis of 15 tick species infesting cattle across six South African provinces, contributing to a deeper understanding of their diversity and ecological roles. These findings align with and expand on previous research, offering new insights into tick-host interactions and the epidemiology of tick-borne diseases.

The morphological traits of *Amblyomma hebraeum*, the most prevalent species identified, are consistent with prior studies, particularly in its long mouthparts and ornate scutum [52,53]. However, this study provides additional data on sexual dimorphism, such as the unique posterior angle of the female scutum, which was not emphasized in earlier works. Such detailed descriptions refine identification criteria and offer valuable information for understanding the reproductive morphology of this species, which directly influences its population dynamics and vector competence [54,55].

*Haemaphysalis silacea*, a less-studied species, exhibited a flat body structure and distinct capitulum morphology, traits highlighted in this study as critical for its identification. This study adds to these observations by documenting unique setae patterns, underscoring the importance of morphological characteristics where molecular tools are less accessible. Ticks of the *Hyalomma* genus, specifically *Hyalomma truncatum* and *Hyalomma rufipes*, displayed robust body structures and elongated mouthparts, consistent with findings by Guglielmone and Nava [56] and Damian, Damas [57]. The distinct reddish-brown coloration of *H. rufipes*, along with its scapular grooves, aligns with previously reported descriptions [57,58]. Notably, the co-occurrence of *H. truncatum* and *H. rufipes* raises questions about interspecies interactions and competition. *Ixodes pilosus*, typically associated with smaller mammals, was identified in cattle in this study, extending its known host range and ecological niche. This rounded-body tick, with shorter mouthparts, contrasts morphologically with *Hyalomma* species and is consistent with descriptions by Nava, Gerardi [59] and Zhang, Liu [60]. Its potential host flexibility may influence its role in disease transmission, a subject that warrants further exploration.

The *Rhipicephalus* genus, which includes species such as *Rhipicephalus microplus*, *Rhipicephalus decoloratus*, and *Rhipicephalus evertsi evertsi*, displayed significant morphological diversity. For example, *R. microplus*, characterized by its reddish-brown scutum and hypostome teeth arranged in 4 + 4 rows, aligns with observations by Low, Tay [61] and Balinandi, Chiţimia-Dobler [62]. Similarly, *R. decoloratus*, identified by its pale yellow translucent scutum, corroborates studies that emphasize its adaptability to humid environments [48,63]. *Rhipicephalus evertsi evertsi*, a species with robust body structures and medium-length hypostomes, highlights its role as a vector for pathogens affecting livestock. The morphological distinctions provided in this study, such as densely punctuated scutums and convex, beady eyes, enhance species differentiation and align with prior reports [52,57].

The study’s findings also reveal important insights into tick–host interactions. The morphological and molecular evidence indicates host flexibility in some species, such as *Ixodes pilosus* and *Rhipicephalus evertsi evertsi*, which may facilitate the cross-species transmission of pathogens. This underscores the importance of understanding these interactions for managing tick-borne diseases in both livestock and wildlife [63,64,65].

### 4.3. Analysis of Molecular Characterization and Phylogenetics of Tick Species

The molecular characterization of ticks using markers such as 16S rRNA has significantly advanced our understanding of tick taxonomy, diversity, and evolutionary relationships. In this study, the use of 16S rRNA highlighted the genetic similarities and divergences within and among tick species, a finding that aligns with previous studies. For instance, Elhelw, Elhariri [66] and Wei, Guo [67] demonstrated the importance of 16S rRNA in resolving cryptic species complexes, particularly in genera like *Rhipicephalus*, where morphological distinctions are often insufficient. Similarly, the strong bootstrap support in phylogenetic clusters observed in our analysis supports the findings of Tagoe [68], who emphasized the genetic differentiation shaped by geographical and ecological factors.

The genetic diversity within tick species, particularly in genera such as *Amblyomma* and *Hyalomma*, reveals both intraspecific similarities and interspecific distinctions. Our findings on *Amblyomma hebraeum* showed high genetic similarity among populations, consistent with the low intraspecific genetic distances reported by Khan, Shehla [69]. However, distinct genetic clusters within species like *Hyalomma truncatum* and *Ixodes pilosus*, shaped by geographical isolation, mirror the observations of Cerqueira, Santos [70] and Hornok [71]. These findings underline the influence of ecological variables and host availability in shaping the genetic structure of tick populations.

Molecular markers also play a pivotal role in understanding the vector potential of ticks. The study identified *Amblyomma hebraeum* as a significant vector for pathogens such as *Rickettsia* and *Anaplasma*, findings supported by Horak, Golezardy [72] and Alghamdi, Low [73]. Similarly, the phylogenetic clustering of *Hyalomma rufipes* and *Hyalomma truncatum* aligns with their documented role in transmitting Crimean-Congo haemorrhagic fever, as described by Perveen, Muzaffar [74] and Cerqueira, Santos [70]. These findings highlight the critical need for molecular tools in disease ecology, particularly in identifying and monitoring potential vectors.

The genetic diversity observed in *Rhipicephalus microplus* and *Rhipicephalus sanguineus* has implications for acaricide resistance and vector control strategies. Our results, which revealed clustering patterns among resistant populations, align with the findings of Damian, Damas [57] and Seo, Kim [75], who emphasized the importance of genetic markers in monitoring resistance. These insights are critical for developing targeted interventions, as different genetic lineages may exhibit varied responses to acaricide treatments.

### 4.4. Implications for Tick Control and Disease Management

The widespread distribution of ticks in South Africa underlines the urgent need for integrated control strategies tailored to the ecological and biological characteristics of each species. The high prevalence of *Amblyomma hebraeum* and *Rhipicephalus evertsi evertsi* in tropical and subtropical regions suggests that these areas should be prioritized for intervention. Integrated pest management approaches, combining the judicious use of acaricides with biological control methods such as entomopathogenic fungi, can significantly reduce tick populations [76]. The presence of *Hyalomma rufipes*, a key vector for CCHF, highlights the need for enhanced public health surveillance and awareness campaigns to mitigate zoonotic risks. Surveillance systems should monitor both tick populations and the pathogens they carry, enabling early detection of outbreaks and timely implementation of control measures [77]. Additionally, the genetic characterization of tick populations can inform control strategies by identifying acaricide resistance patterns and potential pathogen reservoirs. Molecular techniques for pathogen screening, integrated with traditional morphological methods, are crucial for effective vector surveillance and management [78].

### 4.5. Future Directions and Recommendations

This study underscores the need for further research into the ecological dynamics of tick populations. The integration of molecular markers such as COI and intergenic spacer regions with ecological and morphological data will provide a more comprehensive understanding of tick biodiversity and their roles in pathogen transmission. Expanding pathogen screening efforts to include emerging zoonotic agents will also enhance our ability to predict and mitigate the risks of tick-borne diseases.

## 5. Conclusions

This study provides a detailed analysis of tick species infesting cattle in South Africa, offering critical insights into their diversity, distribution, and genetic relationships. By integrating morphological and molecular techniques, it highlights the importance of comprehensive identification strategies for effective tick management and disease prevention. Future research should focus on expanding molecular analyses, incorporating pathogen screening, and developing sustainable control frameworks that address the ecological and epidemiological challenges posed by tick populations.

## Figures and Tables

**Table 1:** Tick species distribution with counts and percentages across sampled provinces.

Species	Limpopo	Kwazulu Natal	Eastern Cape	Mpumalanga	Gauteng	Free State	Total
*A. hebraeum*	575 (30.1%)	1030 (53.8%)	259 (13.5%)	12 (0.6%)	37 (1.9%)	0 (0.0%)	1913
*H. silacea*	0 (0.0%)	0 (0.0%)	17 (100.0%)	0 (0.0%)	0 (0.0%)	0 (0.0%)	17
*H. truncatum*	9 (34.6%)	13 (50.0%)	0 (0.0%)	3 (11.5%)	1 (3.8%)	0 (0.0%)	26
*H. marginatum rufipes*	22 (11.8%)	43 (23.1%)	0 (0.0%)	78 (41.9%)	1 (0.5%)	42 (22.6%)	186
*I. pilosus*	0 (0.0%)	0 (0.0%)	7 (70.0%)	3 (30.0%)	0 (0.0%)	0 (0.0%)	10
*R. decoloratus*	0 (0.0%)	108 (26.3%)	302 (73.7%)	0 (0.0%)	0 (0.0%)	0 (0.0%)	410
*R. evertsi*	194 (38.0%)	80 (15.7%)	52 (10.2%)	25 (4.9%)	125 (24.5%)	34 (6.7%)	510
*R. exophthalmos*	0 (0.0%)	5 (62.5%)	3 (37.5%)	0 (0.0%)	0 (0.0%)	0 (0.0%)	8
*R. glabroscutatus*	0 (0.0%)	5 (100.0%)	0 (0.0%)	0 (0.0%)	0 (0.0%)	0 (0.0%)	5
*R. microplus*	109 (29.9%)	239 (65.5%)	1 (0.3%)	16 (4.4%)	0 (0.0%)	0 (0.0%)	365
*R. appendiculatus*	0 (0.0%)	35 (77.8%)	1 (2.2%)	0 (0.0%)	0 (0.0%)	9 (20.0%)	45
*R. simus*	0 (0.0%)	3 (75.0%)	1 (25.0%)	0 (0.0%)	0 (0.0%)	0 (0.0%)	4
*R. gertrudae*	0 (0.0%)	0 (0.0%)	0 (0.0%)	0 (0.0%)	0 (0.0%)	11 (100.0%)	11
*R. sanguineus*	0 (0.0%)	1 (25.0%)	0 (0.0%)	0 (0.0%)	3 (75.0%)	0 (0.0%)	4
**Total**	909 (25.9%)	1562 (44.5%)	643 (18.3%)	137 (3.9%)	167 (4.8%)	96 (2.7%)	3514

**Table 2:** Maximum likelihood estimate of substitution.

From\To	A	T	C	G
**A**	-	9.5226	2.9154	**5.8889**
**T**	9.5226	-	**5.8889**	2.9154
**C**	9.5226	**19.2352**	-	2.9154
**G**	**19.2352**	9.5226	2.9154	-

**Table 3:** BLAST analysis results for hard tick nucleotide sequences. The percentages indicate the range of identity and coverage between the query sequences and the reference strains. Identity% represents the similarity between the nucleotide sequences of the query and the reference strain, while Covering% indicates the proportion of the query sequence that aligns with the reference sequence.

Tick Species	Identity%	Coverage%	Reference Strains of Tick Species
***A. hebraeum***	98.79–99.76	96–99	KY457513.1
***H. truncatum***	94.8–97.28	94–100	LC634545.1
***R. appendiculatus***	94.8–100	94	KC503257.1
***R. decoloratus***	97.64–99.76	99	LC634555.1
***R. evertsi evertsi***	99–99.5	94–97	LC634571.1
***R. microplus***	99.74–100	94–98	LC634561.1
***R. simus***	97.27–97.28	94–95	LC634554.1

**Table 4:** Pairwise genetic distance matrix of 16S sequences among hard tick specimens. The genetic distance matrix highlights pairwise comparisons between sampled species, with yellow cells indicating a genetic distance of the same species.

	*A. hebraeum*	*A. hebraeum*	*A. hebraeum*	*A. hebraeum. KY457513.1*	*Hae. silacea*	*Hae. silacea*	*Hae. silacea*	*H. marginatum*	*H. marginatum*	*H. marginatum*	*H. rufipes*	*H. truncatum*	*H. truncatum*	*H. truncatum*	*H. truncatum. LC634545.1*	*I. pilosus*	*I. pilosus*	*I. pilosus*	*R. appendiculatus*	*R. appendiculatus*	*R. appendiculatus*	*R. appendiculatus. KC503257.1*	*R. decoloratus*	*R. decoloratus*	*R. decoloratus*	*R. decoloratus. LC634555.1*	*R. evertsi*	*R. evertsi*	*R. evertsi*	*R. evertsi. evertsi. LC634571.1*	*R. gertrudae*	*R. gertrudae*	*R. gertrudae*	*R. glabroscutatus*	*R. glabroscutatus*	*R. glabroscutatus*	*R. microplus*	*R. microplus*	*R. microplus*	*R. microplus. LC634561.1*	*R. simus*	*R. simus*	*R. simus*	*R. simus. LC634554.1*
*A. hebraeum*																																												
*A. hebraeum*	0.00																																											
*A. hebraeum*	0.00	0.00																																										
*A. hebraeum. KY457513.1*	0.00	0.01	0.00																																									
*Hae. silacea*	0.15	0.15	0.15	0.16																																								
*Hae. silacea*	0.15	0.15	0.15	0.16	0.00																																							
*Hae. silacea*	0.17	0.17	0.17	0.17	0.01	0.01																																						
*H. marginatum*	0.21	0.21	0.21	0.22	0.16	0.16	0.17																																					
*H. marginatum*	0.21	0.21	0.21	0.22	0.16	0.16	0.17	0.00																																				
*H. marginatum*	0.21	0.21	0.21	0.22	0.16	0.16	0.17	0.00	0.00																																			
*H. rufipes*	0.22	0.22	0.22	0.22	0.16	0.16	0.17	0.00	0.00	0.00																																		
*H. truncatum*	0.22	0.22	0.22	0.23	0.16	0.16	0.18	0.03	0.03	0.03	0.03																																	
*H. truncatum*	0.22	0.22	0.22	0.23	0.16	0.16	0.17	0.03	0.03	0.03	0.03	0.00																																
*H. truncatum*	0.22	0.22	0.22	0.23	0.16	0.16	0.17	0.03	0.03	0.03	0.03	0.01	0.01																															
*H. truncatum. LC634545.1*	0.24	0.24	0.24	0.23	0.18	0.18	0.19	0.04	0.04	0.04	0.04	0.02	0.02	0.01																														
*I. pilosus*	0.20	0.20	0.20	0.22	0.18	0.18	0.19	0.25	0.25	0.25	0.26	0.27	0.27	0.27	0.30																													
*I. pilosus*	0.22	0.22	0.22	0.24	0.20	0.20	0.21	0.28	0.28	0.28	0.29	0.29	0.29	0.29	0.32	0.02																												
*I. pilosus*	0.21	0.21	0.21	0.23	0.19	0.19	0.20	0.27	0.27	0.27	0.28	0.28	0.28	0.28	0.31	0.01	0.02																											
*R. appendiculatus*	0.23	0.23	0.23	0.25	0.18	0.18	0.19	0.17	0.17	0.17	0.17	0.17	0.18	0.18	0.19	0.27	0.29	0.28																										
*R. appendiculatus*	0.23	0.23	0.23	0.24	0.18	0.18	0.19	0.17	0.17	0.17	0.18	0.18	0.18	0.18	0.19	0.27	0.29	0.28	0.00																									
*R. appendiculatus*	0.23	0.23	0.23	0.25	0.18	0.18	0.19	0.17	0.17	0.17	0.17	0.17	0.18	0.18	0.19	0.27	0.29	0.28	0.00	0.00																								
*R. appendiculatus. KC503257.1*	0.24	0.24	0.24	0.24	0.19	0.19	0.20	0.18	0.18	0.18	0.18	0.18	0.19	0.19	0.19	0.28	0.31	0.29	0.00	0.00	0.00																							
*R. decoloratus*	0.21	0.21	0.21	0.21	0.17	0.17	0.18	0.18	0.18	0.18	0.18	0.18	0.18	0.17	0.18	0.27	0.29	0.28	0.13	0.13	0.13	0.13																						
*R. decoloratus*	0.19	0.19	0.19	0.20	0.16	0.16	0.17	0.16	0.16	0.16	0.16	0.16	0.16	0.16	0.17	0.25	0.27	0.26	0.12	0.12	0.12	0.12	0.01																					
*R. decoloratus*	0.19	0.19	0.19	0.20	0.16	0.16	0.17	0.16	0.16	0.16	0.16	0.16	0.16	0.16	0.17	0.25	0.27	0.26	0.12	0.12	0.12	0.12	0.01	0.00																				
*R. decoloratus. LC634555.1*	0.21	0.21	0.21	0.20	0.17	0.17	0.17	0.17	0.17	0.17	0.17	0.17	0.17	0.17	0.17	0.26	0.29	0.27	0.12	0.12	0.12	0.11	0.01	0.00	0.00																			
*R. evertsi*	0.21	0.21	0.21	0.22	0.18	0.18	0.19	0.14	0.14	0.14	0.15	0.15	0.15	0.14	0.15	0.28	0.30	0.29	0.09	0.09	0.09	0.09	0.09	0.08	0.08	0.08																		
*R. evertsi*	0.21	0.21	0.21	0.22	0.18	0.18	0.19	0.14	0.14	0.14	0.15	0.15	0.15	0.14	0.15	0.28	0.30	0.29	0.09	0.09	0.09	0.09	0.09	0.08	0.08	0.08	0.00																	
*R. evertsi*	0.21	0.21	0.21	0.22	0.18	0.18	0.19	0.14	0.14	0.14	0.15	0.15	0.15	0.14	0.15	0.28	0.30	0.29	0.09	0.09	0.09	0.09	0.09	0.08	0.08	0.08	0.00	0.00																
*R. evertsi. evertsi. LC634571.1*	0.21	0.21	0.21	0.21	0.18	0.18	0.18	0.15	0.15	0.15	0.15	0.15	0.15	0.15	0.15	0.29	0.31	0.30	0.10	0.10	0.10	0.09	0.08	0.07	0.07	0.08	0.01	0.01	0.01															
*R. gertrudae*	0.22	0.22	0.22	0.23	0.20	0.20	0.20	0.16	0.16	0.16	0.16	0.15	0.15	0.15	0.16	0.25	0.27	0.27	0.09	0.09	0.09	0.10	0.13	0.12	0.12	0.12	0.10	0.10	0.10	0.10														
*R. gertrudae*	0.22	0.22	0.22	0.23	0.20	0.20	0.20	0.16	0.16	0.16	0.16	0.15	0.15	0.15	0.16	0.25	0.27	0.26	0.09	0.09	0.09	0.10	0.13	0.12	0.12	0.12	0.10	0.10	0.10	0.10	0.00													
*R. gertrudae*	0.22	0.22	0.22	0.23	0.20	0.20	0.20	0.16	0.16	0.16	0.16	0.15	0.15	0.15	0.16	0.25	0.27	0.27	0.09	0.09	0.09	0.10	0.13	0.12	0.12	0.12	0.10	0.10	0.10	0.10	0.00	0.00												
*R. glabroscutatus*	0.21	0.21	0.21	0.22	0.17	0.17	0.18	0.15	0.15	0.15	0.15	0.13	0.13	0.13	0.15	0.23	0.25	0.24	0.07	0.07	0.07	0.08	0.12	0.11	0.11	0.12	0.08	0.08	0.08	0.08	0.07	0.07	0.07											
*R. glabroscutatus*	0.20	0.20	0.20	0.22	0.17	0.17	0.18	0.15	0.15	0.15	0.15	0.13	0.13	0.13	0.15	0.23	0.25	0.24	0.07	0.07	0.07	0.07	0.12	0.11	0.11	0.12	0.08	0.08	0.08	0.08	0.07	0.07	0.07	0.00										
*R. glabroscutatus*	0.21	0.21	0.21	0.22	0.17	0.17	0.18	0.15	0.15	0.15	0.15	0.13	0.13	0.13	0.15	0.23	0.25	0.24	0.07	0.07	0.07	0.07	0.12	0.11	0.11	0.12	0.08	0.08	0.08	0.08	0.07	0.07	0.07	0.00	0.00									
*R. microplus*	0.19	0.19	0.19	0.20	0.16	0.16	0.17	0.13	0.13	0.13	0.13	0.13	0.13	0.13	0.14	0.25	0.27	0.27	0.12	0.12	0.12	0.13	0.08	0.07	0.07	0.08	0.09	0.09	0.09	0.08	0.11	0.11	0.11	0.10	0.10	0.10								
*R. microplus*	0.19	0.19	0.19	0.20	0.16	0.16	0.17	0.13	0.13	0.13	0.13	0.13	0.13	0.13	0.14	0.25	0.27	0.27	0.12	0.12	0.12	0.13	0.08	0.07	0.07	0.08	0.09	0.09	0.09	0.08	0.11	0.11	0.11	0.10	0.10	0.10	0.00							
*R. microplus*	0.19	0.19	0.19	0.20	0.16	0.16	0.17	0.13	0.13	0.13	0.13	0.13	0.13	0.13	0.14	0.25	0.27	0.27	0.12	0.12	0.12	0.13	0.08	0.07	0.07	0.08	0.09	0.09	0.09	0.08	0.11	0.11	0.11	0.10	0.10	0.10	0.00	0.00						
*R. microplus. LC634561.1*	0.20	0.20	0.20	0.20	0.17	0.17	0.18	0.13	0.13	0.13	0.13	0.13	0.14	0.14	0.14	0.27	0.29	0.28	0.12	0.13	0.12	0.12	0.08	0.08	0.08	0.08	0.09	0.09	0.09	0.08	0.12	0.12	0.12	0.10	0.10	0.10	0.00	0.00	0.00					
*R. simus*	0.22	0.22	0.22	0.23	0.20	0.20	0.20	0.15	0.15	0.15	0.16	0.15	0.15	0.14	0.16	0.25	0.27	0.26	0.08	0.08	0.08	0.08	0.13	0.12	0.12	0.12	0.10	0.10	0.10	0.10	0.02	0.03	0.02	0.08	0.08	0.08	0.11	0.11	0.11	0.12				
*R. simus*	0.22	0.22	0.22	0.23	0.19	0.19	0.20	0.15	0.15	0.15	0.15	0.15	0.15	0.14	0.16	0.25	0.27	0.26	0.08	0.08	0.08	0.08	0.13	0.11	0.11	0.12	0.10	0.10	0.10	0.09	0.03	0.03	0.03	0.07	0.07	0.08	0.11	0.11	0.11	0.11	0.00			
*R. simus*	0.22	0.22	0.22	0.23	0.19	0.19	0.20	0.15	0.15	0.15	0.15	0.15	0.15	0.14	0.16	0.25	0.27	0.26	0.08	0.08	0.08	0.08	0.13	0.11	0.11	0.12	0.10	0.10	0.10	0.09	0.03	0.03	0.03	0.07	0.07	0.08	0.11	0.11	0.11	0.11	0.00	0.00		
*R. simus. LC634554.1*	0.24	0.24	0.24	0.24	0.21	0.21	0.22	0.17	0.17	0.17	0.17	0.16	0.16	0.16	0.17	0.27	0.29	0.28	0.10	0.09	0.10	0.09	0.13	0.12	0.12	0.12	0.10	0.10	0.10	0.09	0.03	0.03	0.03	0.07	0.07	0.07	0.12	0.12	0.12	0.12	0.03	0.03	0.03	

## Data Availability

The data supporting the reported results are available in the GenBank repository. The sequences generated in this study have been deposited under the accession numbers PP789312–PP789494 and can be accessed at https://www.ncbi.nlm.nih.gov/genbank (accessed on 18 November 2024).
